# Demographics and clinical outcomes of autistic children and adolescents receiving psychiatric in-patient care in the Thames Valley from 2019 to 2024: retrospective cohort study

**DOI:** 10.1192/bjo.2026.12039

**Published:** 2026-07-24

**Authors:** Samuel Lloyd-White, Rohan Borschmann, Alice Rorke, Eloise Stark, Gillian Combe

**Affiliations:** Thames Valley Tier 4 CAMHS Provider Collaborative, Oxford Health NHS Foundation Trust, Oxford, UK; Health Service and Population Research Department, Institute of Psychiatry, Psychology & Neuroscience, King’s College London, London, UK; Better Health & Care Hub, King’s College London, London, UK; Department of Psychiatry, University of Oxford, Oxford, UK; Centre for Adolescent Health, Murdoch Children’s Research Institute and Royal Children’s Hospital, Melbourne, Australia; Melbourne School of Psychological Sciences, Faculty of Medicine, Dentistry and Health Sciences, University of Melbourne, Melbourne, Australia

**Keywords:** Autism, in-patient, outcomes, child and adolescent mental health services

## Abstract

**Background:**

Many autistic adolescents receive in-patient psychiatric care from services that are ill-equipped to meet neurodivergent needs. However, the outcomes for autistic adolescents accessing in-patient care remain poorly understood.

**Aims:**

To document the demographics and clinical outcomes of autistic adolescents referred to in-patient psychiatric services over a 5-year period and compare these with those of their allistic peers accessing the same services.

**Method:**

We conducted a retrospective cohort study involving all adolescents (aged 12–17 years inclusive) referred for in-patient psychiatric care through the Thames Valley Provider Collaborative between 1 April 2019 and 31 March 2024. Clinical characteristics and outcomes of referrals for autistic and allistic adolescents were collected and summarised from routine service data. Inferential statistics (including *t*-tests and chi-squared tests) were used to compare characteristics between the autistic and allistic groups.

**Results:**

Autistic adolescents were more likely than their allistic peers to be referred in an emergency (24.6 *v*. 16.6%) and for risk management (53.7 *v*. 33.5%). Autistic adolescents without an eating disorder had longer average length of stay than their peers (146.1 *v.* 97.5 days) and were more likely to be discharged to more secure settings following admission (12.9 *v*. 7.7%). Emerging evidence from routine outcome measures suggests less positive progress during in-patient care and greater impairment among autistic adolescents at both admission and discharge.

**Conclusions:**

There are important differences in outcomes from psychiatric in-patient admissions between autistic and allistic adolescents. Greater work is needed to understand these differences and the factors influencing treatment success for autistic adolescents.

In the UK, rates of diagnosed autism in childhood have increased significantly in the past 20 years.^1^ It is currently estimated that approximately 3% of children and adolescents in the UK aged 10–17 years have a diagnosis of autism without a co-occurring intellectual disability.^2^ During this time, there has been a corresponding increase in the proportion of autistic children and adolescents seeking support from child and adolescent mental health services (CAMHS). An estimated 10% of CAMHS patients are autistic, a marked overrepresentation relative to the general population.^3^ This number rises further still to those accessing in-patient mental healthcare, with 13% of patients in in-patient mental healthcare diagnosed with autism.^4^ Additionally, many more young people are awaiting diagnostic assessment as services specialising in the assessment and treatment of neurodevelopmental conditions such as autism struggle to keep pace with demand.^5^

The National Strategy for Autistic Children, Young People and Adults 2021–2026^6^ recognises that autistic people, particularly children and adolescents, are too often being admitted to in-patient settings after struggling to access appropriate care in the community to prevent a mental health crisis. Despite current UK Mental Health Act legislation^7^ stipulating that autism is a ‘mental disorder’ for which a person can be detained, there is a growing policy direction to amend this^8^ and to reduce the rate of in-patient psychiatric admissions for autistic young people.^9–11^ Despite ongoing concern about the experiences and outcomes of autistic children and adolescents accessing in-patient services, the rate of psychiatric hospital admission is significantly higher for autistic adolescents than for adolescents with other developmental or psychiatric conditions.^12–14^ Autistic adolescents also typically have longer lengths of stay than non-autistic adolescents (hereafter referred to as allistic).^12–14^

Caring for autistic adolescents in in-patient care can involve unique challenges, as the in-patient environment and brief interventions are often inappropriate for young people with significant communication, sensory and social differences.^15–17^ Specialist support to make reasonable adjustments to care and interagency collaboration to minimise the use and duration of in-patient care is required.^11^ Effective and accepted support often requires nuanced, idiosyncratic understanding in formulation and person-centred treatment goals.^18^ Support often also requires contextual interventions and joined-up, systemic working^19^ that includes exploring the possibility of neurodivergence within the wider family.^20^ Delivering this within an in-patient setting can be a challenge because of training and skills deficits.^6^

A limited number of studies have demonstrated positive outcomes associated with in-patient mental health admissions in mixed samples including children and adolescents with neurodevelopmental conditions.^21,22^ However, little is known about the clinical and demographic characteristics, experiences and clinical outcomes of autistic children and adolescents accessing in-patient psychiatric services. To address this notable evidence gap, in this study we aimed to (a) examine the demographics, clinical characteristics and clinical outcomes of autistic children and adolescents referred for in-patient psychiatric services over a 5-year period through one provider collaborative in the South of England; and (b) compare these with those of their allistic peers (i.e. those without an autism diagnosis) referred during the same period.

## Method

### Setting and participants

In England, ‘provider collaboratives’ are networks of specialised mental health, intellectual disability and autism service providers working together to improve the care pathways of patients in their local areas. Importantly, they have responsibility for the budget and clinical pathways for their local population, both of which previously sat under the remit of NHS England, a key organisation within the National Health Service (NHS) in England, established to oversee and improve healthcare services.

The Thames Valley Provider Collaborative (TVPC) is a network of in-patient and highly specialist mental health facilities in the South of England responsible for providing psychiatric treatment to all people under the age of 18 years who require in-patient psychiatric care in Gloucestershire, Swindon, Wiltshire, Oxfordshire, Buckinghamshire, Berkshire, Bath and North-East Somerset. The TVPC serves a population of 3.7 million people – representing 5.5% of the UK population^23^ – and includes a population of approximately 800 000 people aged <18 years. Service provision within the TVPC comprised two general adolescent units (GAUs) for the duration of the data collection period and one further GAU that closed and was remodelled as a day service. Psychiatric intensive care unit (PICU) provision was available throughout the data collection period; however, the initial provider and setting closed down during the period and was replaced by a new facility operated by a new provider. These changes did result in a fluctuation in capacity for in-area admissions throughout the data collection period.

Systematic routine data collection regarding referrals within the TVPC began on 1 April 2019. In the current study, we conducted a retrospective cohort analysis of all referrals received by the TVPC in the period 1 April 2019 to 31 March 2024, inclusive.

Where referrals were accepted for admission, the majority (83.7%) were admitted to the in-patient services within the TVPC outlined above. The remainder of the referrals accepted for admission were sent to providers outside of the TVPC, either because of service constraints at the time of referral or the need for specialist provision not available within the TVPC (e.g. secure, specialist intellectual disability or specialist eating disorder settings).

### Data collection

CAMHS services in England are arranged in a tier system, whereby Tier 1 represents primary care services, Tiers 2 and 3 are specialised community services, and Tier 4 services are in-patient or alternative-to -admission services. Referrals were made through a single point of access (SPA), using the nationally standardised referral form for Tier 4 CAMHS by senior clinicians. Data were extracted into a central database from information provided by referring clinicians and TVPC SPA staff screened referrals for data completeness, requesting additional information from referrers where missing data was identified. Only referrals with 100% data completeness were accepted. Where a young person’s referral was accepted and they were admitted, ongoing routine outcome data were collected by the TVPC SPA team from the admitting unit. Upon discharge, additional information was extracted from the discharge summary.

The information used in the current study was de-identified data collected in routine clinical practice for service delivery. As such, in accordance with NHS Health Research Authority guidance outlining that such data may be used for research without formal ethics approval from a research ethics committee,^24^ consent from patients was not sought or required and the study was not deemed to require such approval.

### Demographic and clinical data

All referrals included key demographic (age, gender, ethnicity) and clinical (diagnosis/es, type of service(s) requested, urgency of request, current location) information. As referrals were only accepted with 100% data completeness, there were no missing data. For referrals that were accepted and subsequently admitted, additional clinical information regarding duration of admission and discharge destination was collected. Confirmed diagnoses of autism made during admission or recommendations for assessment based on suspected autism were systematically documented – this group of young people were not included in the autistic cohort for data analysis. As routine outcome measures were only systematically collected and collated across the commissioned units within the provider collaborative in 2023–2024, data collected before this point were excluded to prevent skewing of the data.

The measures used across the TVPC were all measures included in the Child Outcomes Research Consortium database (www.corc.uk.net) and are widely used in routine clinical practice nationally.

- Child Global Assessment Scale (CGAS):^25^ a clinician-report measure of general functioning. Scores range from 0 to 100, with lower scores representing lower levels of functioning.
- Revised Child Anxiety and Depression Scale (RCADS):^26^ a self-report questionnaire of anxiety and depression symptoms. Raw scores are converted into standardised *T*-scores for the appropriate age and gender, with a *T*-score of 65 representing the clinical cut-off. Higher scores indicate greater severity of symptoms. Although a parent-report version is also available, this was not routinely used in the current study and therefore not included. Only RCADS total scores were submitted to the TVPC SPA for data collation, therefore subscales are not reported.
- Health of the Nation Outcome Scales for Children and Adolescents (HoNOSCA):^27^ a clinician and self-reported measure of general health and social functioning. Scores range from 0 to 52, with higher scores indicating greater impairment. Only clinician-reported data were systematically collected and therefore self-report scores are not included.

### Data analysis

Data from both the autistic and allistic samples were analysed. The autistic sample included young people who had a confirmed diagnosis of autism on referral and excluded young people who received a diagnosis of autism or suspected autism during their admission. All referrals received within the data collection period were included in analyses of referral characteristics, and analyses of discharge characteristics were conducted on a subgroup of the data-set who were both admitted and discharged within the data collection period. Categorical data (e.g. demographic and clinical characteristics) were described with counts and percentages, and continuous data (e.g. length of stay, CGAS scores) were described with means and standard deviations. For categorical data, proportions were calculated and chi-square analyses were used to compare between the two groups, with *φ* and Cramer’s *V* used to calculate effect sizes. For continuous variables, independent samples *t*-tests were used to compare the difference between variables, with Cohen’s *d* used to calculate effect sizes. To compare outcomes between groups at admission and discharge, a two-way mixed analysis of variance was undertaken with diagnosis (autistic versus allistic) as the between-participants factor and time point (admission versus discharge) as the within-participants factor. Partial *η*^2^ was used to calculate effect sizes. *Post hoc* comparisons were undertaken with a Tukey’s correction applied. All analyses were conducted with IBM SPSS Statistics version 22 (for Windows; IBM Corp, New York, USA; https://www.ibm.com/products/spss-statistics).

### Study approval

This study was approved by the Oxford Health NHS Foundation Trust Quality and Audit Team and deemed not to require approval by an ethics committee.

## Results

**Fig. 1:**
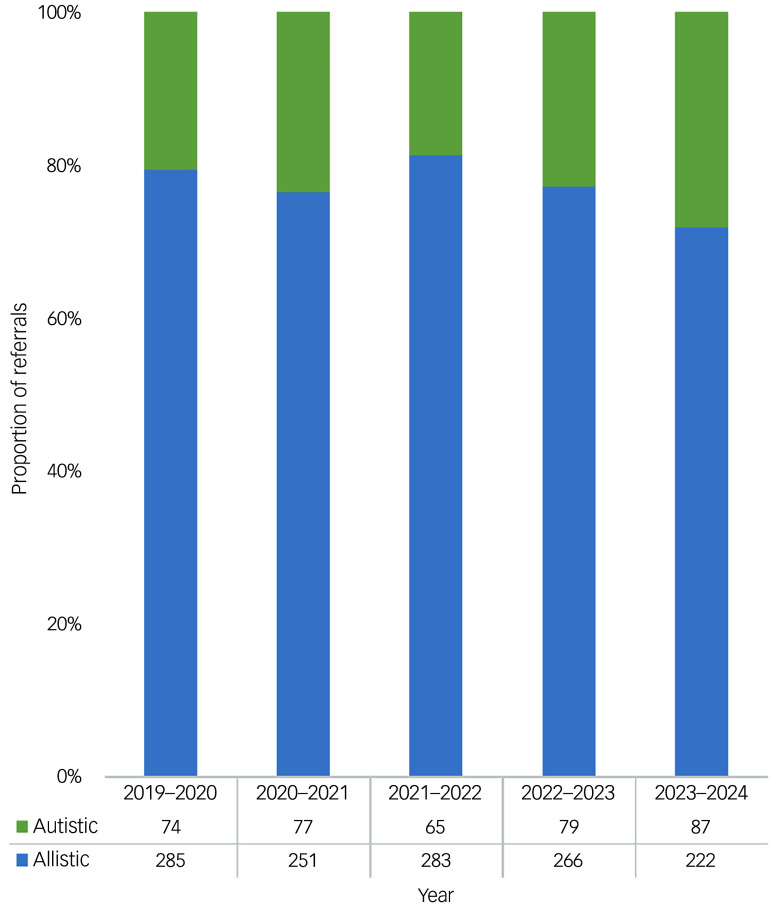
Proportion of allistic versus autistic adolescent referrals over time.

Tables 1–3 summarise the clinical and demographic information for the autistic and allistic cohorts/samples. Most referrals were from community services within the TVPC catchment area, but a small proportion of referrals (26.9%) were out-of -area requests for specialist services or transfers within in-patient services. The total number of adolescents referred within the time frame for data collection was 1689, of whom 382 (22.6%) had a confirmed diagnosis of autism on referral. The proportion of adolescents with a confirmed diagnosis of autism on referral remained largely static within the data collection period, ranging from 23.5% in 2019–2020 to 18.7% in 2020–2021 (see Fig. 1).

**Table 1:** Demographic information Table 1 long description.

Patient group	Gender, *n* (%)					Ethnicity, *n* (%)						Median age in years at referral (IQR)
	Male	Female	Trans male	Trans female	Non-binary	White	Black	Asian	Mixed	Other	Not stated	
Allistic	149 (11)	1130 (86)	24 (2)	1 (<1)	3 (<1)	1101 (84)	28 (2)	52 (4)	58 (4)	45 (3)	23 (2)	16 (1)
Autistic	84 (22)	271 (71)	19 (5)	2 (1)	6 (2)	321 (84)	4 (1)	13 (3)	31 (8)	4 (1)	9 (2)	15 (2)
Overall	233 (14)	1401 (83)	43 (3)	3 (<1)	9 (1)	1422 (84)	32 (2)	65 (4)	89 (5)	49 (3)	32 (2)	16 (2)

IQR, interquartile range.

**Table 2:** Referral information Table 2 long description.

Patient group	Type of referral*				Type of unit*								
	Repatriation	Routine	Urgent	Emergency	Day patient	General adolescent unit	Hospital at home	Intellectual disability/autism	Low secure	Medium secure	Other	Psychiatric intensive care	Specialist eating disorder
Allistic, *n* (%)	21 (2)	374 (29)	695 (53)	217 (17)	143 (11)	521 (40)	65 (5)	4 (<1)	29 (2)	2 (<1)	1 (<1)	128 (10)	414 (32)
Autistic, *n* (%)	9 (2)	88 (23)	191 (50)	94 (25)	31 (8)	187 (49)	8 (2)	12 (3)	18 (5)	6 (2)		68 (18)	52 (14)
Overall, *n* (%)	30 (2)	462 (27)	886 (52)	311 (18)	174 (10)	708 (42)	73 (4)	16 (1)	47 (3)	8 (<1)	1 (<1)	196 (12)	466 (28)

* Indicates significant difference between autistic and allistic individuals at the *p* < 0.05 level.

**Table 3:** Clinical information Table 3 long description.

Patient group	Admissions, *n* (%)	Mean length of stay in days (s.d.)	Eating disorder referral, *n* (%)
Allistic	912 (70)	115 (108)	724 (55)
Autistic	251 (66)	143 (250)	139 (36)
Overall	1163 (69)	121 (150)	863 (51)

### Referral characteristics

Of the 1307 referrals received for allistic adolescents, 69.8% (*n* = 912) were accepted for admission, compared with the 65.7% (*n* = 251) of referrals for autistic people who were accepted for admission during the same period. The proportion of referrals accepted for admission did not vary significantly between groups (*χ* ^2^ (1) = 2.26, *p* = 0.113).

There was a significant association between the presence of an autism diagnosis and the type of unit requested (*χ* ^2^ (8) = 109.34, *p* < 0.001), reason for referral (risk management versus treatment) (*χ* ^2^ (5) = 62.24, *p* < 0.001), classification of the referral (routine, urgent, emergency) (*χ* ^2^ (3) = 15.12, *p* < 0.001), use of the Mental Health Act (*χ* ^2^ (1) = 48.46, *p* < 0.001) and location on referral (*χ* ^2^ (4) = 25.58, *p* < 0.001).

*Post hoc* analyses indicated that autistic adolescents were considerably more likely to be referred to a PICU than allistic adolescents (15.1 *v*. 8.9%, respectively). Autistic adolescents were also considerably more likely to be referred for risk management (53.7 *v*. 33.5%) and considerably less likely to be referred for treatment (30.9 *v*. 51.3%), compared with allistic adolescents.

**Fig. 2:**
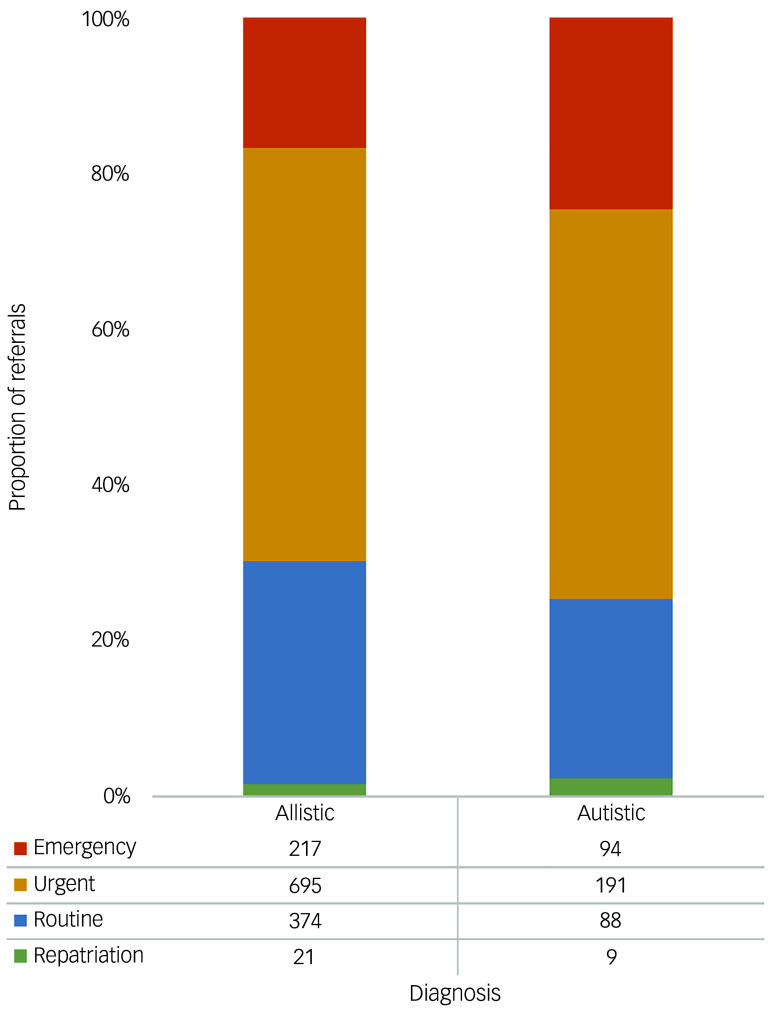
Classification of referrals for allistic versus autistic adolescents.

Referrals for autistic adolescents (24.6%) were more likely to be classed as emergency referrals than those for allistic adolescents (16.6%), with a corresponding reduction in the proportion of routine referrals (23.0 *v*. 28.6%) (see Fig. 2). Additionally, compared with allistic adolescents, a greater proportion of referrals received for autistic adolescents were detained under the Mental Health Act (47.9 *v*. 28.8%) and a greater proportion (13.4 *v*. 7.5%) listed the location on referral as a place of safety following detention under Section 136 of the Mental Health Act. Section 136 is used when somebody presents in crisis in the community and is transported to an approved place of safety by police for assessment.

### Discharge characteristics

Although the average length of stay for autistic adolescents (mean 142.6 days, s.d. 250.0) was slightly longer than for allistic adolescents (mean 114.9 days, s.d. 106.7), this difference was not significant (*t*(271.6) = −1.07, *p* = 0.089). A significant proportion of admissions to Tier 4 services are for the treatment of eating disorders, and these admissions are typically of longer duration, which may skew figures in the allistic population if there is an uneven distribution of eating disorders referrals within the two groups. Of all referrals accepted for admission, 55% of allistic young people had an identified eating disorder on referral compared with 36.3% of autistic young people.

**Fig. 3:**
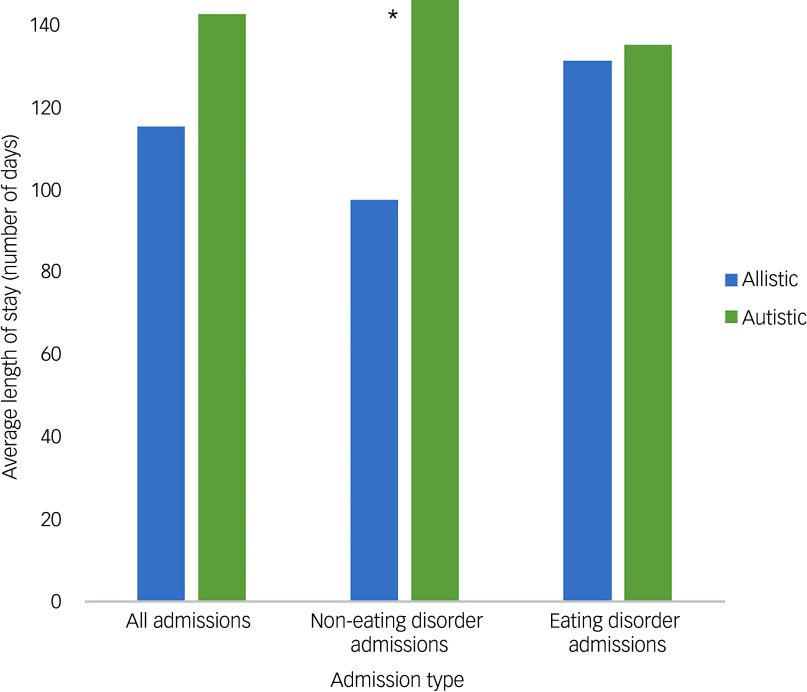
Average length of stay for allistic versus autistic adolescents. * Indicates significant difference at the *p* < 0.05 level.

To address this potential confounding variable, a comparison of non-eating disorder admissions only was undertaken. The disparity in average length of stay increased further, to 146.1 days (s.d. 290.6) for autistic adolescents and 97.5 days (s.d. 112.0) for allistic adolescents, and reached statistical significance (*t*(187.7) = −2.12, *p* = 0.036) (see Fig. 3).

As referral severity is also not evenly distributed between autistic and allistic individuals, it is possible that greater acuity on admission may result in a longer admission. Subsequent analysis of the difference in length of stay for autistic and allistic young people stratified by referral severity indicated that, for both routine (168.5 *v*. 131.2 days) and urgent (139.5 *v*. 112.5 days) referrals, autistic young people stayed in hospital for longer on average. This discrepancy was not evident for emergency referrals (88.0 *v*. 90.7 days), indicating that referral acuity was not the primary driver for greater length of stay for autistic individuals.

**Fig. 4:**
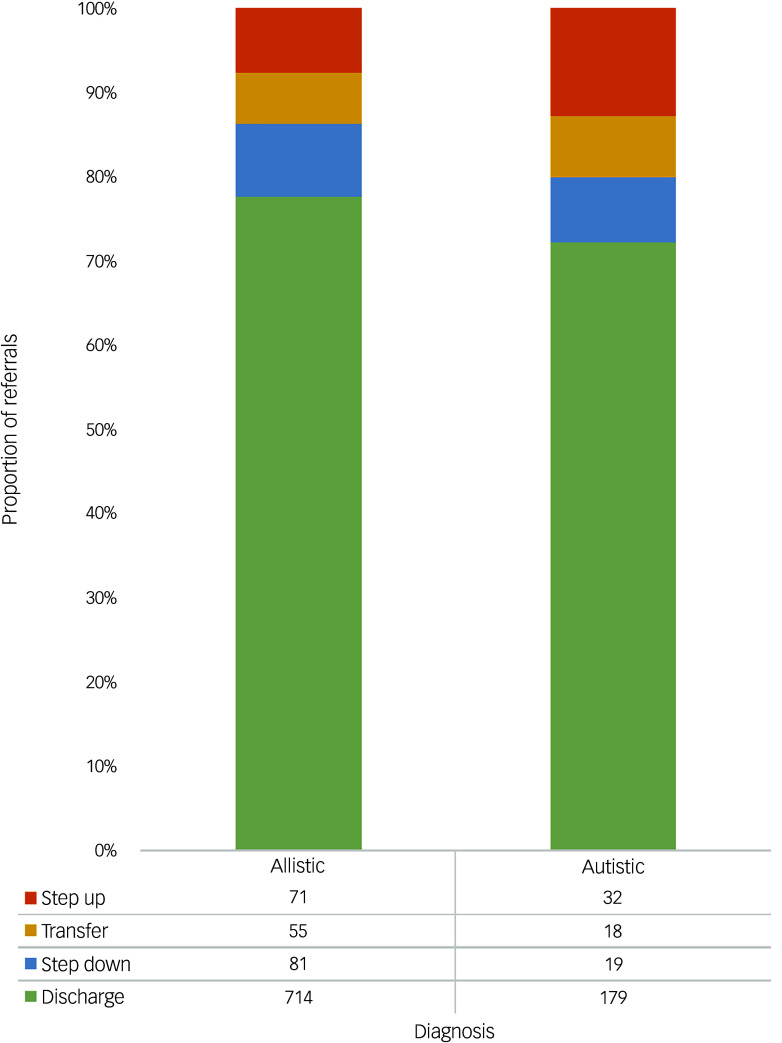
Summary of discharge destination for allistic versus autistic patients.

On discharge, a greater proportion of autistic adolescents (12.9%) were transferred to a unit with a higher level of security (e.g. GAU to PICU) than allistic adolescents (7.7%), although again this difference did not reach statistical significance (*χ* ^2^ (4) = 7.74, *p* = 0.101). See Fig. 4 for a summary.

Further analysis was completed to understand the relationship between referral severity, autism diagnosis and discharge destination, to address the potential for greater referral acuity in the autistic population as a confounding variable. This revealed that there was a consistent pattern of divergence between autistic and allistic individuals for routine (11.4 *v*. 7.7%, respectively) and urgent referrals (12.4 *v*. 7.1%, respectively). This divergence was not present for emergency referrals, suggesting that referral severity was not driving the increased likelihood of step-up for autistic individuals.

**Fig. 5:**
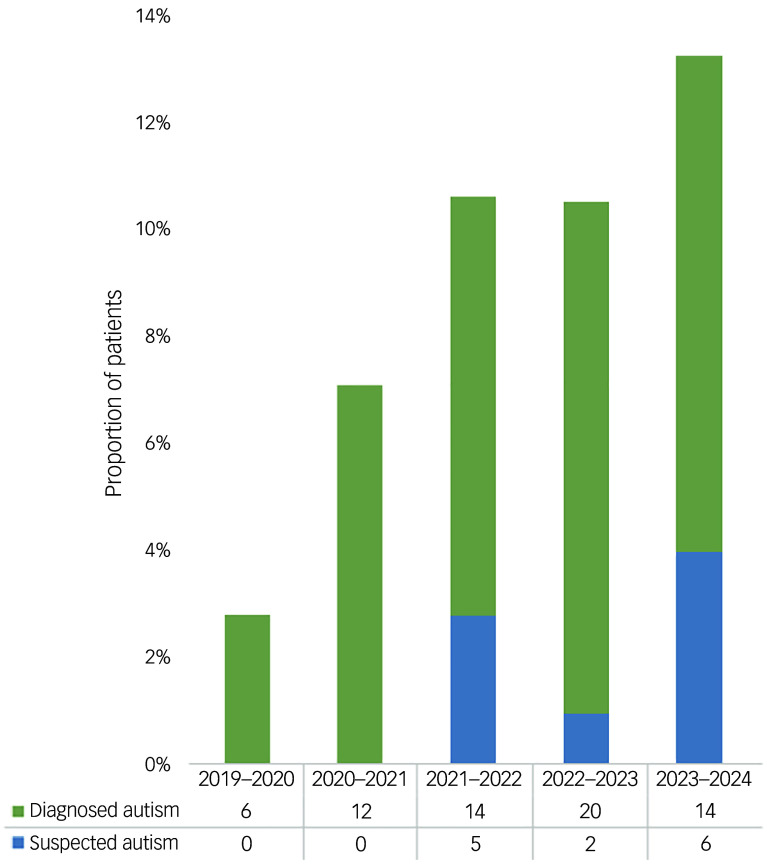
Patients who received a diagnosis of autism during their admission over time.

The proportion of adolescents who received a diagnosis of autism during admission was 7.1%. This was a steadily increasing trend, and in the final 2 years of data collection the percentage was over 9%. In addition, there was an increasing proportion of adolescents who did not receive a diagnosis, but were suspected of being autistic, and recommendation for assessment was made on the discharge summary (see Fig. 5 for details).

### Outcome measures

#### CGAS

**Fig. 6:**
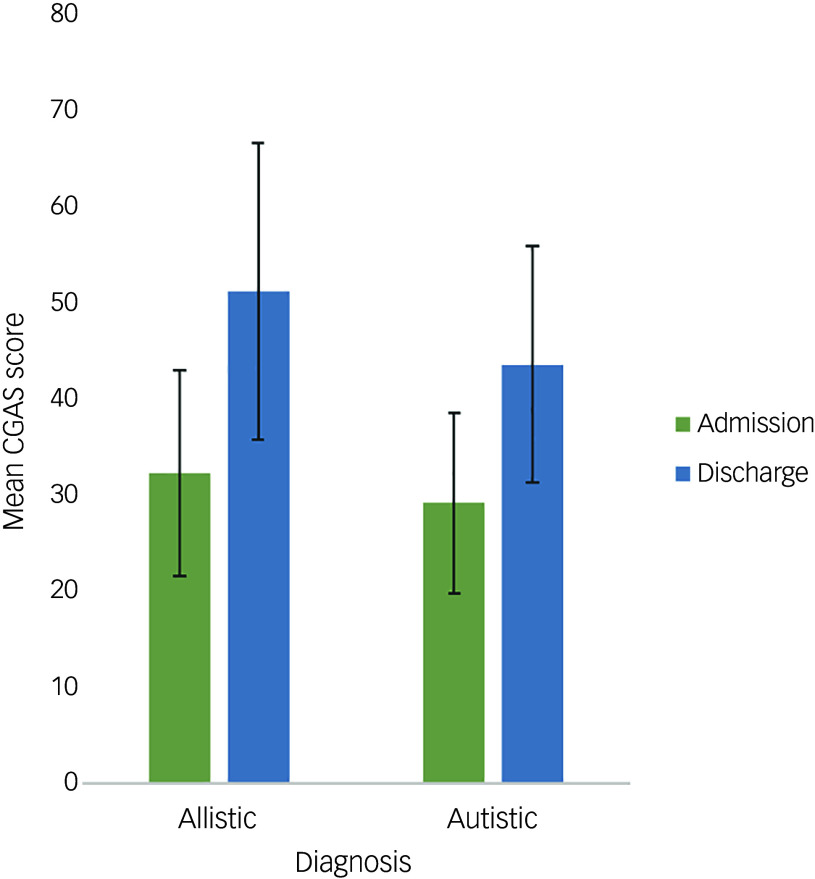
Child Global Assessment Scale (CGAS) scores on admission and discharge.

There were 337 paired samples for allistic adolescents and 110 for autistic adolescents. There was no significant interaction between diagnosis and time point (*F*(1,253) = 3.00, *p* = 0.085). There were significant main effects for both autism (*F*(1,253) = 10.9, *p* = 0.001, partial *η* ^2^ = 0.041) and time point (*F*(1,253) = 209.65, *p* < 0.001, partial *η* ^2^ = 0.453). Autistic adolescents had a marginally lower CGAS score on admission (mean 29.1, s.d. 9.4) compared with their allistic peers (mean 32.2, s.d. 10.9), but this was not significant. On discharge, scores for autistic adolescents had improved (mean 43.5, s.d. 12.3), but less so than for allistic adolescents (mean 51.1, s.d. 15.1) (see Fig. 6).

#### RCADS

**Fig. 7:**
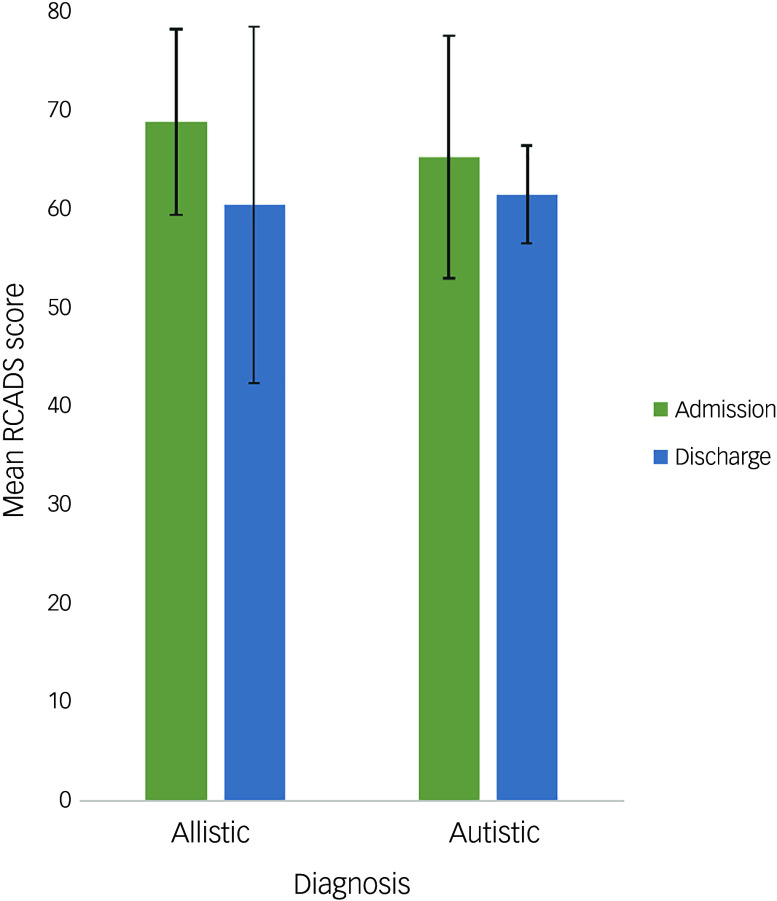
Revised Child Anxiety and Depression Scale (RCADS) scores on admission and discharge.

There were a total of 25 paired samples for allistic adolescents and 14 paired samples for autistic adolescents. There was no significant interaction between autism and time point (*F*(1,22) = 0.03, *p* = 0.869). There was a significant main effect for time point (*F*(1,22) = 6.23, *p* = 0.021, partial *η* ^2^ = 0.221), but not for autism. Autistic adolescents scored marginally lower on depression and anxiety on admission (mean 65.3, s.d. 12.3) compared with their allistic peers (mean 68.8, s.d. 9.4). On discharge, scores for autistic adolescents had improved slightly (mean 61.5, s.d. 5.0), but less so than for allistic adolescents (mean 60.4, s.d. 18.0) (see Fig. 7).

#### HoNOSCA

There were a total of 156 paired samples for allistic adolescents and 48 paired samples for autistic adolescents. There was a significant interaction between autism and time point (*F*(1,215) = 5.44, *p* = 0.021 partial *η* ^2^ = 0.025). There were significant main effects for both autism (*F*(1,215) = 4.05, *p* = 0.046, partial *η* ^2^ = 0.018) and time point (*F*(1,215) = 66.44, *p* < 0.001, partial *η* ^2^ = 0.236). *Post hoc* comparisons with a Tukey’s correction revealed no significant differences in scores at admission (*t*(215) = 0.61, *p* = 0.930); however, at discharge, scores differed significantly (*t*(215) = 2.64, *p* = 0.044).

**Fig. 8:**
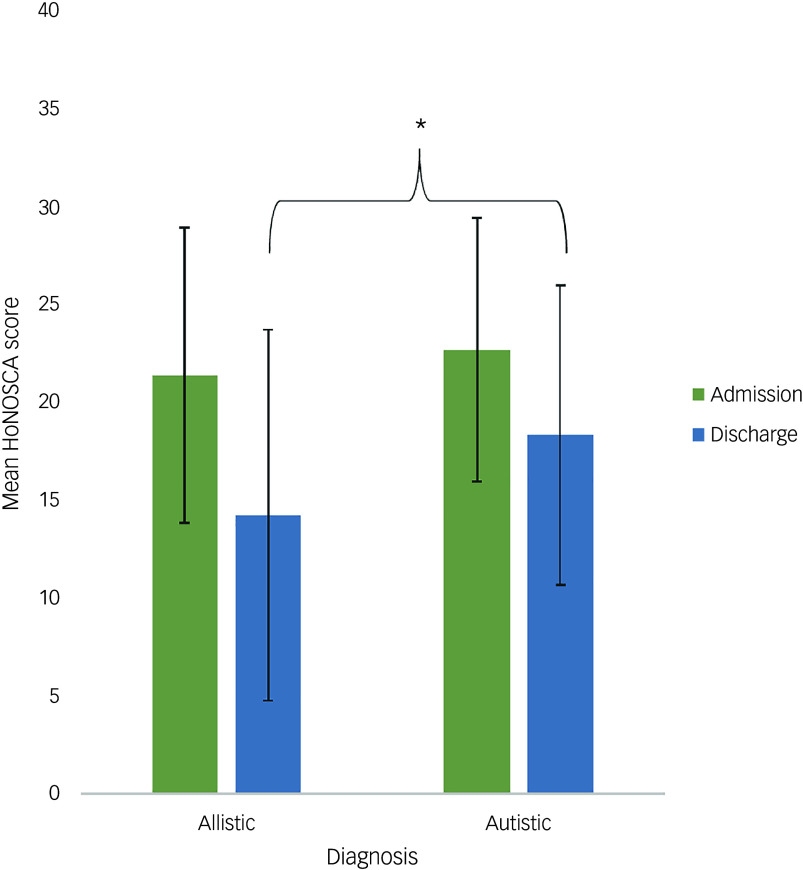
Health of the Nation Outcomes Scale for Children and Adolescents (HoNOSCA) scores on admission and discharge. * Indicates significant difference at the *p* < 0.05 level.

Autistic adolescents scored marginally higher on admission (mean 22.7, s.d. 6.7) compared with allistic peers (mean 21.4, s.d. 7.4). On discharge, scores for autistic adolescents had improved slightly (mean 18.3, s.d. 7.6), but less so than for allistic adolescents (mean 14.3, s.d. 9.3) (see Fig. 8).

Stratified change scores for autistic and allistic individuals according to referral acuity were reviewed, and revealed a consistent pattern of difference across the measures. For the HoNOSCA, autistic individuals’ scores changed by 4.5 fewer points for routine referrals and 4.2 fewer points for urgent referrals when compared with their allistic referrals. Change scores for emergency referrals were not calculated because of insufficient sample size.

A similar pattern was evident for CGAS, with 4.2 points fewer change for routine referrals. For urgent and emergency referrals, the difference was only one point. Differences between autistic and allistic young people on the RCADS were minimal across all referral severities. This indicates that referral severity alone did not account for differences in outcome between autistic and allistic adolescents.

## Discussion

This study examined the demographics, clinical characteristics and clinical outcomes of autistic children and adolescents referred for in-patient psychiatric services in the South of England, and compared these with those of their peers without an autism diagnosis who were referred during the same period. Our findings illustrate a continuing picture of overrepresentation of autistic adolescents in mental health in-patient settings. It is noteworthy, however, that although the community picture is one of a continually increasing prevalence of diagnosed autism,^1^ the trend in the current sample remained largely static at approximately one in five referrals.

This difference in trend may result, in part, from increased efforts and policy directives over the study period to actively promote alternatives to admission in all but the most severe circumstances.^28^ Of particular importance nationally is the implementation of ‘blue light’ Care, Education and Treatment Reviews for all young people with autism at high risk of admission, and the use of the Dynamic Support Register to monitor those at risk of admission and provide additional support.^29^ Locally, there is an understanding of the need to provide intensive community provision through crisis and intensive home treatment teams and a recently established hospital at home service, which provides a Tier 4 alternative-to -admission service for those with significantly complex needs relating to their autism.

It is noteworthy that the current study found an increasing trend of autism being diagnosed during in-patient admissions and a greater still increase in the identification of traits warranting further assessment upon discharge. Although this trend may reflect increasing struggles with waiting time for community assessments, and the potential burnout associated with having undiagnosed autism,^30^ it may also reflect increased clinical awareness of the features of autism, and therefore a reflection of greater detection rather than greater prevalence.

The current study also demonstrated an increased likelihood of referrals for autistic adolescents at the point of crisis, with a greater emphasis on risk management and treatment in more restrictive settings, such as PICUs, as well as greater use of the Mental Health Act for compulsory assessment and/or treatment. This may reflect those with less severe needs being actively supported to avoid admission; however, the subgroup of autistic adolescents who present with high-risk behaviours warrants further exploration to understand unique risk factors and unmet needs within this population.^31^

The average lengths of stay in the current study were shorter than some of the statistics outlined in the 2017 Transforming Care report,^11^ commissioned in response to concerns about long-stay in-patient admissions for autistic people in the UK. There is a clear indication, however, that hospital stays are typically longer for autistic adolescents than for their allistic peers. It is important to further understand the reasons for this, whether it is related to the acuity and complexity of presentations or issues with delayed discharges related to developing packages of care. Our findings suggest that admissions for autistic adolescents that followed routine or urgent referrals are more likely to be transferred to more secure services, and outcome measures show modest improvement overall, although less than their allistic peers even when referral severity is accounted for. This indicates that autistic adolescents may face a number of potential challenges or barriers to good clinical outcomes in in-patient settings.

In addition, there are ongoing national shortages in the UK of appropriate regulated social care placements for adolescents with complex mental health difficulties.^32^ Such placements are typically resource-intensive and require collaboration between the health, social care and education sectors, which can result in significant delays to discharge planning. Autistic people are more likely to require support of this nature on discharge, because of their more complex and multifaceted needs.^33^

### Strengths and limitations

The current study draws upon a large data-set of 1689 referrals over a 5-year period, representing a considerable sample size. The findings represent an attempt to understand the characteristics and outcomes of autistic adolescents in in-patient settings that have previously been understudied. The sample size for routine outcome measures was considerably smaller; therefore, these findings should be interpreted with caution. In addition, the data gathered relied predominantly on clinician report, limiting the inclusion of patient and carer perspectives. Although the RCADS has demonstrated good validity and reliability in autistic young people without intellectual disability,^34^ there are no currently published studies demonstrating the validity of CGAS and HoNOSCA in this population.

The outcome measures used in the current study do not, by design, distinguish between the underlying factors contributing to functional impairment.^25–27^ Consequently, CGAS and HoNOSCA scores should be interpreted cautiously, as they reflect overall global functioning, which may be influenced by autistic traits as well as mental health, emotional and other psychosocial factors.^35^

Although there is ongoing debate regarding the sensitivity of routine outcome measures in in-patient settings for both autistic and allistic young people,^36^ this is beyond the scope of the current study. Nevertheless, careful consideration of meaningful and person-centred outcomes in in-patient settings remains essential to accurately measure clinical progress.

The documentation of diagnoses on referral forms was inconsistent and unvalidated; therefore, further detail relating to comorbidities, such as attention-deficit hyperactivity disorder, was excluded from data analyses because of concerns about data quality. Young people receiving Tier 4 care often present with complex, multiple comorbidities. This is a potential limitation, and the presence of comorbidity is a potential confounding variable that warrants further exploration in future research. Information regarding diagnosis of autism (or suspected autism) during admission was extracted from written discharge summaries, and therefore there may be some missing or incomplete data. It is possible that the stark trend of increasing rates of suspected autism reflects increased documentation rather than an increased prevalence. Although the current study does highlight some trends regarding outcomes and characteristics for adolescents with autism accessing in-patient care, it was beyond the scope of the current study to explore in more detail factors that predict treatment outcome for autistic adolescents in in-patient settings, to further support with clinical decision-making and refinement of treatment.

As the current study draws mainly on retrospective categorical data only from those referred and accepted, it is difficult to draw firm conclusions about the specific iatrogenic harm of an in-patient admission for autistic young people or the specific additional risks of poor outcomes for autistic young people. It may be that referrals made under the Mental Health Act and/or for risk management are associated with poorer outcomes in isolation; however, the greater proportion of autistic young people within these categories is still of clinical significance, and warrants further investigation. Further study comparing outcomes for autistic young people who access alternatives to admission and in-patient care would also further our understanding of specific risks of hospital admission for this group.

### Clinical implications

There is clear evidence of need for careful consideration of the potential for less positive outcomes of admission for autistic adolescents when compared with their allistic peers. Autistic adolescents may stay longer in hospital, show less improvement on routine outcome measures and are more likely to require a step up to a higher level of security, even when controlling for acuity on admission. This further strengthens the current policy direction of utilising specialist alternative-to -admission services that can offer a more tailored and autism-informed approach. Where an admission is unavoidable, it is important that all agencies work collaboratively to minimise admission length wherever possible, and early discussion of ongoing care needs is essential.

Further work is required to examine how best to adapt in-patient settings to meet the needs of autistic adolescents to minimise potential harms and maximise the chance of achieving therapeutic benefits, including the optimisation of any pharmacological treatment required.^37,38^

### Research implications

Further research is required to understand those factors that predict outcomes at all stages of the patient journey for autistic adolescents. Greater formulation of the unmet needs of those adolescents who present in crisis is required to support service planning and, for those who do access in-patient services with a positive outcome, understanding the factors that predict treatment success would support further adaptation of in-patient services. For those who do access alternative-to -admission services, a comparison of outcomes with peers who access in-patient care would be of great benefit.

The current study highlighted an increasing trend of diagnoses being made during admission. At present, there are no clear guidelines for best practice in conducting assessments specifically in in-patient psychiatric settings. Further work to understand the training needs of clinicians working in in-patient settings, and unique considerations when undertaking assessment in this context, is required to ensure a robust process and optimise in-patient treatment outcomes for these highly vulnerable adolescents.

## Data Availability

Data for the current study were extracted from a locally held database used for routine clinical and performance monitoring. Consent to share with third parties has not been gained and so it is not possible to make the data publicly available.

## Table 1 Long description

The table presents demographic information for autistic and allistic patient groups, including gender, ethnicity, and median age at referral. It has 3 rows and 11 columns. The columns are labeled as follows: Patient group, Gender (Male, Female, Trans male, Trans female, Non-binary), Ethnicity (White, Black, Asian, Mixed, Other, Not stated), and Median age in years at referral (IQR). The row labels are Allistic, Autistic, and Overall. Row 1: Allistic, Male 149 (11 percent), Female 1130 (86 percent), Trans male 24 (2 percent), Trans female 1 (<1 percent), Non-binary 3 (<1 percent), White 1101 (84 percent), Black 28 (2 percent), Asian 52 (4 percent), Mixed 58 (4 percent), Other 45 (3 percent), Not stated 23 (2 percent), Median age in years at referral 16 (1). Row 2: Autistic, Male 84 (22 percent), Female 271 (71 percent), Trans male 19 (5 percent), Trans female 2 (1 percent), Non-binary 6 (2 percent), White 321 (84 percent), Black 4 (1 percent), Asian 13 (3 percent), Mixed 31 (8 percent), Other 4 (1 percent), Not stated 9 (2 percent), Median age in years at referral 15 (2). Row 3: Overall, Male 233 (14 percent), Female 1401 (83 percent), Trans male 43 (3 percent), Trans female 3 (<1 percent), Non-binary 9 (1 percent), White 1422 (84 percent), Black 32 (2 percent), Asian 65 (4 percent), Mixed 89 (5 percent), Other 49 (3 percent), Not stated 32 (2 percent), Median age in years at referral 16 (2).

Navigate back to Table 1.

## Table 2 Long description

The table presents referral information for allistic and autistic patient groups, detailing the type of referral and the type of unit. It has 7 columns and 4 rows including header. Column headers are: Patient group, Type of referral, Type of unit, Repatriation, Routine, Urgent, Emergency, Day patient, General adolescent unit, Hospital at home, Intellectual disability/autism, Low secure, Medium secure, Other, Psychiatric intensive care, Specialist eating disorder. Row labels are: Allistic, n (%), Autistic, n (%), Overall, n (%). Row 1: Allistic, n (%), 21 (2), 374 (29), 695 (53), 217 (17), 143 (11), 521 (40), 65 (5), 4 (1), 29 (2), 2 (1), 1 (1), 128 (10), 414 (32). Row 2: Autistic, n (%), 9 (2), 88 (23), 191 (50), 94 (25), 31 (8), 187 (49), 8 (2), 12 (3), 18 (5), 6 (2), 68 (18), 52 (14). Row 3: Overall, n (%), 30 (2), 462 (27), 886 (52), 311 (18), 174 (10), 708 (42), 73 (4), 16 (1), 47 (3), 8 (1), 1 (1), 196 (12), 466 (28).

Navigate back to Table 2.

## Table 3 Long description

The table presents clinical and demographic information for autistic and allistic patient groups. It has three rows and four columns. The columns are labeled ‘Patient group’, ‘Admissions, n (%)’, ‘Mean length of stay in days (s.d.)’, and ‘Eating disorder referral, n (%)’. The rows are labeled ‘Allistic’, ‘Autistic’, and ‘Overall’. Row 1: Allistic, 912 (70), 115 (108), 724 (55). Row 2: Autistic, 251 (66), 143 (250), 139 (36). Row 3: Overall, 1163 (69), 121 (150), 863 (51).

Navigate back to Table 3.
